# Cryptogenic recurrent spontaneous intracranial epidural hematoma: A case report and literature review

**DOI:** 10.3389/fneur.2023.1123108

**Published:** 2023-03-16

**Authors:** Min Xu, Ya Xue, Xiaofeng Chao, Zhenglou Chen, Yunjiang Wang, Xuqi Huo, Xiang Ji, Hongshen Wang

**Affiliations:** ^1^Department of Neurosurgery, The Sixth Affiliated Hospital of Nantong University, Yancheng, Jiangsu, China; ^2^Department of Neurosurgery, Yancheng Third People's Hospital, Yancheng, Jiangsu, China; ^3^Department of Neurosurgery, The Yancheng School of Clinical Medicine of Nanjing Medical University, Yancheng, Jiangsu, China; ^4^Department of Neurosurgery, The Affiliated Yancheng Hospital of Southeast University, Yancheng, Jiangsu, China; ^5^Department of Neurosurgery, The Second Affiliated Hospital of Xuzhou Medical University, Xuzhou, Jiangsu, China

**Keywords:** spontaneous epidural hematoma, evacuation of epidural hematoma, treatment, ICP, coagulation

## Abstract

**Background:**

Spontaneous epidural hematoma (EDH) has been suggested to be associated with adjacent infective pathologies, dural vascular malformations, extradural metastases, or coagulopathies. Cryptogenic spontaneous EDH is extremely rare.

**Case presentation:**

The present study reports the case of a cryptogenic spontaneous EDH in a young woman following sexual intercourse. She was diagnosed with consecutive EDH at three different sites within a short time. After three timely operations, a satisfactory outcome was achieved.

**Conclusion:**

EDH should be investigated when a young patient develops headaches and shows signs of increased ICP after emotional hyperactivity or hyperventilation. If early diagnosis and surgical decompression can be carried out in time, the prognosis would be satisfactory.

## Introduction

Intracranial epidural hematoma (EDH) is a hematoma that occurs between the inner plate of the skull and the dura mater, which accounts for ~30% of all traumatic intracranial hematoma cases. EDH is mainly caused by a skull fracture after a violent collision of the head, which induces hemorrhage by tearing in the meningeal arteries, the meningeal veins, and the venous sinuses. In contrast, spontaneous EDH is rare and mostly occurs due to adjacent infective pathologies, hematological system diseases, immune system diseases, vascular malformations, and skull metastases, among others.

The occurrence of cryptogenic spontaneous EDH without a specific underlying disease is extremely rare in this category (1, 2). The unidentified primary causes may lead to recurrent episodes of EDH, resulting in severe neurological dysfunction. In this study, we report a case of cryptogenic recurrent spontaneous EDH (three times) in a 22-year-old woman who received three surgical procedures and finally achieved satisfactory outcomes. The literature that focused on the underlying mechanism of spontaneous EDH was further reviewed.

## Case presentation

### First EDH

**Figure 1:**
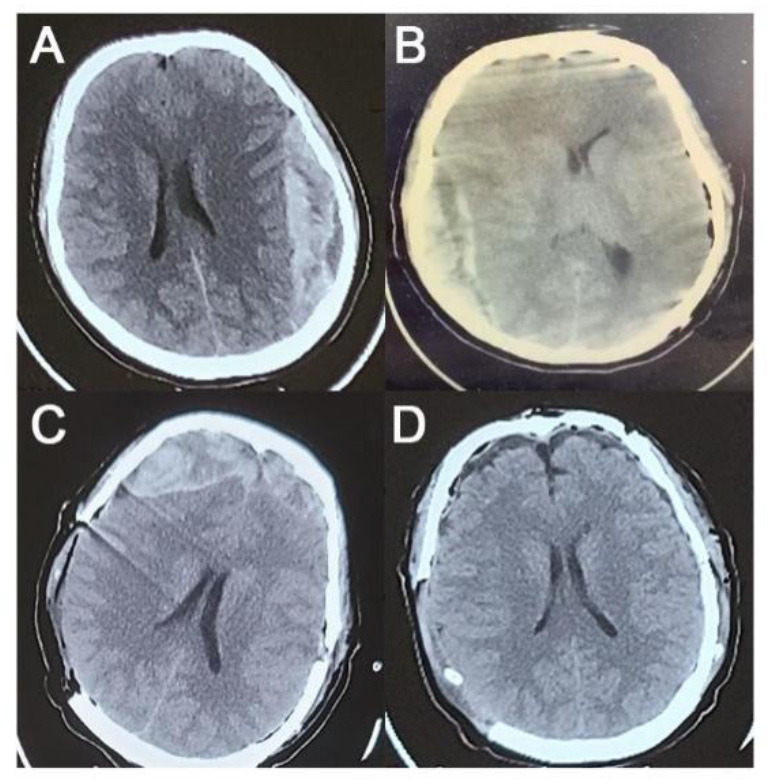
Preoperative and postoperative CT images. **(A)** Preoperative CT. **(B)** CT scan after the first operation. **(C)** CT scan after the second operation. **(D)** CT scan after the third operation.

A 22-year-old woman was admitted with a blunt headache 1 h after intercourse. On general physical examination, the patient was clearly conscious, and her speech was fluent. There was no obvious swelling or injury on the head or face. A neurologic specialist's physical examination revealed that the Glasgow Coma Scale (GCS) score was 15 (eye-opening, 4; motor responsiveness, 6; and verbal performance, 5). There were no obvious positive signs of damage to the nervous system except for headaches and dizziness. An emergent non-enhanced computed tomography (CT) scan (each layer with a 5-mm thickness) revealed a left temporoparietal EDH (the inner and outer diameters were 3 cm; the maximum diameter was >10 cm; the upper and lower diameters were 7.5 cm) with a rightward midline shift of 1 cm. Bone window imaging confirmed no fracture line (Figure 1A). Preoperative coagulation tests showed that all coagulation parameters were within the normal range (Table 1). Past medical history suggested that the patient had undergone a medical abortion 1 month prior, but the specific drug used was unknown.

**Table 1:** Coagulation and blood routine parameters during hospitalization.

**Parameters**	**Normal reference range**	**Preoperative**	**Postoperative day 1**	**Discharge**
**Coagulation parameters**				
Thrombin time (TT), s	16–18	16.6	15.5	15.5
Prothrombin time (PT), s	9–13	12	12.2	11.4
Prothrombin time ratio	0.72–1.24	1.04	1.06	0.99
International Normalized Ratio (INR)	0.8–1.3	1.04	1.06	0.99
Partial prothrombin time, s	20–35	26.4	26.8	26.4
Activated partial thrombin time (APTT), s	23–27	26.4	26.8	26.4
Fibrinogen, g/L	2–4	2.39	2.73	4.37
Fibrinogen degradation products, mg/L	0–5	18.7	5.7	6.4
Antithrombin III activity, %	75–125	84.9	85.5	108.7
D-dimer, mg/L FEU	0–0.55	9.34	1.67	1.87
**Blood routine parameters**				
Erythrocyte, 10^12^/L	3.8–5.1	3.6	2.27	3.45
Hematocrit	0.35–0.45	0.346	0.216	0.325
Hemoglobin, g/L	115–150	114	71	104
Blood platelets, 10^9^/L	125–350	169	151	288
White blood cells, 10^9^/L	3.5–9.5	10.2	12.5	7.01
Thrombocytocrit	0.108–0.271	0.2	0.18	0.3

During preoperative preparation, the patient became increasingly agitated and lost consciousness. Before the administration of anesthesia, the GCS score was 11 (eye opening, 2; motor responsiveness, 5; and verbal performance, 4). Bilateral pupils were equiround with a diameter of 3 mm, and the light reflex disappeared. Two hours after admission, the left temporo-occipital EDH was cleared under general anesthesia in the emergency operating room. No significant fracture lines or EDH-responsible vessels were found during the operation. The intraoperative blood loss was ~300 ml, and no blood transfusion was performed.

### Second EDH

After the first operation, the patient's right pupil was dilated to a diameter of 5.0 mm and the light reflex disappeared, while the patient's left pupil was dilated to a diameter of 3.0 mm and the light reflex disappeared. Immediate CT examination showed right temporo-occipital EDH (the inner and outer diameters were 3 cm; the maximum diameter was >10 cm; the upper and lower diameters were 8.5 cm) with a leftward midline shift of 1 cm (Figure 1B). The right temporo-occipital EDH was removed immediately under general anesthesia. Decompression with flap removal was performed considering the preoperative cerebral hernia and high intraoperative intracranial pressure (ICP). No significant fracture lines or EDH-responsible vessels were found during the operation. The intraoperative blood loss was ~300 ml. During the operation, 3U of red blood cells and 625 ml of plasma were transfused.

### Third EDH

After the second operation, the patient's right pupil was retracted to a diameter of 3.5 mm, and the light reflex was sluggish. The third immediate CT examination showed bilateral frontal EDH (the inner and outer diameters of the right frontal part were 3.5 cm; the hematoma volume was >30 ml; the inner and outer diameters of the left frontal part were >2 cm; the hematoma volume was 20 ml) (Figure 1C). The removal of the bilateral frontal EDH was continued in the emergency operating room. Similar to earlier CT images, no fracture line or EDH-responsible vessels were found during the operation. The intraoperative blood loss was ~ 350 ml, with 200 ml of plasma transfused.

**Figure 2:**
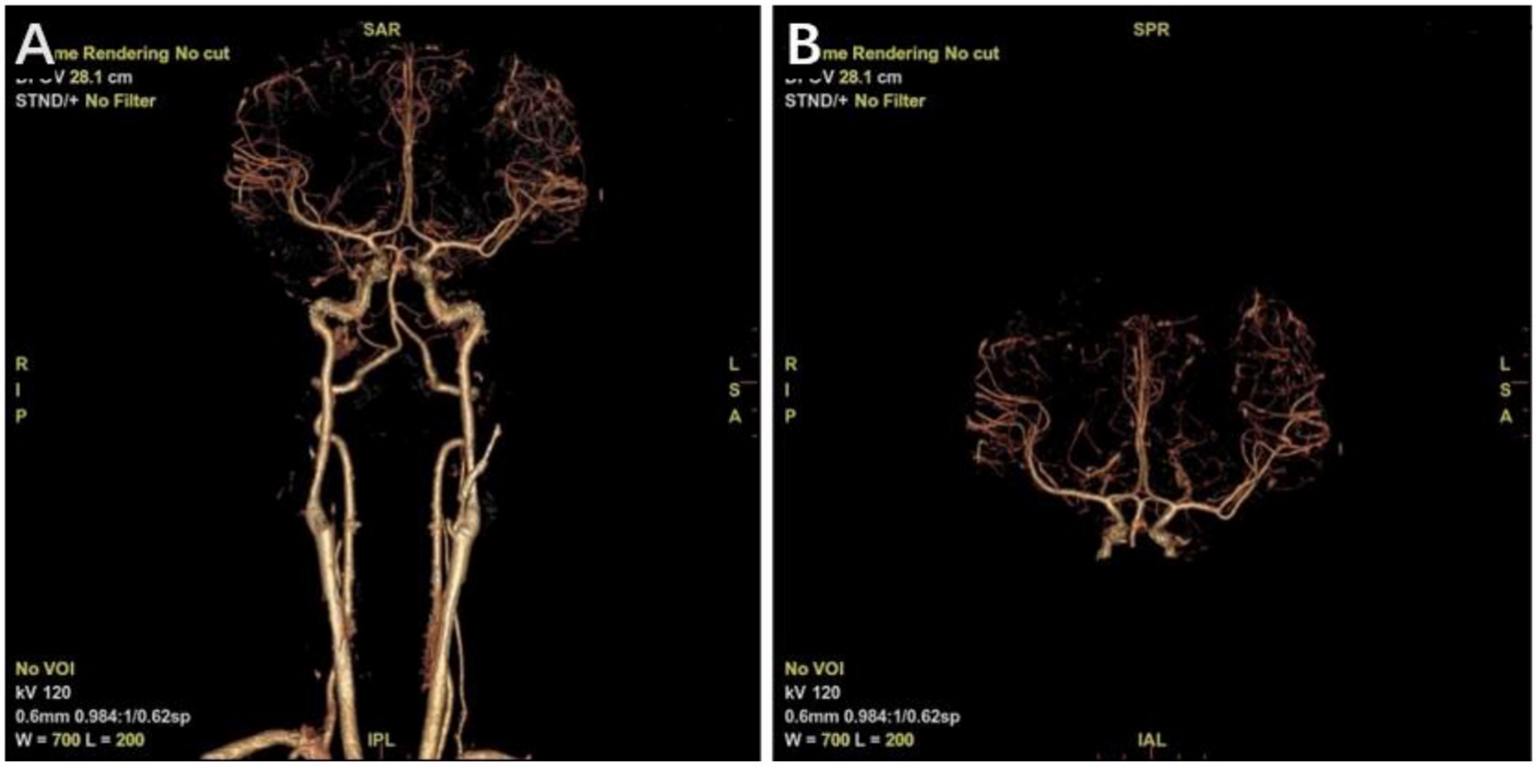
Computed tomography angiography (CTA) at 6 months postoperatively. **(A)** Whole figure. **(B)** Local amplification.

After resuscitation from anesthesia, the patient resumed spontaneous breathing and regained consciousness. The GCS score was 14 (eye opening, 3; motor responsiveness, 6; and verbal performance, 5). The postoperative CT scan showed satisfactory hematoma clearance, and the midline did not shift (Figure 1D). The patient was discharged 2 weeks after surgery with an mRS score of 0. There were no significant abnormalities in the CTA examination in the outpatient clinic 6 months after the operation (Figure 2), and the mRS score was 0.

## Discussion

An epidural hematoma can be classified as traumatic or spontaneous, depending on the presence or absence of violence. Traumatic EDH is mostly caused by skull fractures or lacerations of the meningeal arteries, the meningeal veins, and/or the venous sinuses.

Spontaneous EDH can be divided into primary spontaneous EDH and secondary spontaneous EDH. The former is EDH without relevant underlying diseases. The latter is usually caused by infectious diseases, vascular malformations, blood system diseases, metastases, etc. The patient we reported had no history of trauma, and preoperative and intraoperative examinations ruled out the possible primary underlying diseases mentioned earlier. (The patient had a history of drug abortion 1 month before admission. The specific drug name is unknown. It should be a commonly used, specific drug. However, it has no lasting effect on blood clotting or intracranial pressure. In addition, the patient developed a pelvic mass 2 weeks before admission, which was later surgically confirmed as an ovarian cyst. Moreover, blood coagulation and other indicators on admission are normal, excluding any influence on the incidence.) Therefore, we diagnosed this patient as having cryptogenic spontaneous EDH (primary).

### Cryptogenic spontaneous EDH (primary)

Previous studies reported a total of three cryptogenic spontaneous EDH cases (1–3). Chen et al. proposed that restlessness can induce elevated blood pressure and diffuse microbleeding of the dural vessels, which can stop spontaneously under normal circumstances (1). When patients suffer from persistent irritability accompanied by acute hyperventilation, hypocapnia, and intracranial alkalosis will cause dramatic contraction of the arterial vessels, resulting in decreased cerebral blood flow. These physiological reactions eventually decrease ICP. The dissection of the dura and skull increases the accumulation of microbleeds, eventually forming the cryptogenic spontaneous EDH (1, 4). In our case, we noticed that the patient's first EDH occurred 1 h after intercourse; therefore, we speculated that the underlying mechanism might be similar to the aforementioned hypothesis, that is, an intense physiological reaction causes a sharp drop in ICP and eventually leads to the dissection of the dura from the skull.

Unfortunately, the patient experienced another two consecutive EDHs at different sites during the acute phase after the first surgery. The underlying mechanism of the recurrent EDH may be the sharp drop in ICP after the removal of the hematoma and the dura dissection due to brain tissue displacement. In addition, the dura mater of young people does not adhere closely to the skull, and it may be easier to peel off than that of the elderly.

### Spontaneous EDH with a specific underlying disease (secondary)

Infectious diseases, such as periodontitis and maxillary sinusitis, infect the meningeal arteries and vessels between the skull and diploe by retrograde infection, leading to vascular inflammation. On the one hand, accumulation of inflammatory exudate, pus, and air in the epidural space may cause separation of the dura from the skull. On the other hand, the infiltrating effects of inflammation lead to thinning and increased permeability of the diploic vessel wall, resulting in a breakthrough hematoma in the epidural space (5–7). Dural arteriovenous malformations have been reported as a possible cause of EDH (8). Chen et al. reported a case of spontaneous EDH caused by Langerhans cell histiocytosis (LCH), which was mainly due to osteolytic changes in the skull (9). Over 20 cases of spontaneous EDH caused by sickle cell disease (SCD) have been reported in previous studies. The main mechanism was believed to be the rapid proliferation and expansion of the bone marrow tissue (hematopoietic), which destroys the normal anatomical structure of the skull (10). Some brain metastases (BMS) can invade the dura mater and destroy the adjacent bone even causing coagulation dysfunction, and ultimately leading to the occurrence of EDH (11–13). Complement activation and immune complex deposition in the vascular wall are also involved in the onset of spontaneous EDH (14). These substances will increase the permeability of the blood–brain barrier (BBB) and then stimulate vascular endothelial cells through inflammatory cytokines or autoantibodies. On account of immune injuries, such as cerebral cortex atrophy, cerebral hemorrhage, cerebral infarction, and intracranial arteriovenous multiple sclerosis.

## Conclusion

Cryptogenic spontaneous EDH is a rare condition. EDH should be investigated when a young patient develops headaches and signs of increased ICP after emotional hyperactivity or hyperventilation. If early diagnosis and surgical decompression can be carried out in time, the prognosis will be satisfactory.

## Data availability statement

The raw data supporting the conclusions of this article will be made available by the authors, without undue reservation.

## Ethics statement

The studies involving human participants were reviewed and approved by the Ethics Committee of the Third People's Hospital of Yancheng. Written informed consent to participate in this study was provided by the patients/participants' legal guardian/next of kin. Written informed consent was obtained from the individual's legal guardian/next of kin for the publication of any potentially identifiable images or data included in this article.

## Author contributions

MX, YX, and XC prepared the original draft. ZC, YW, XH, and XJ analyzed the patients' information. HW supervised the entire research process. All authors agreed to be accountable for all aspects of the work in ensuring that questions related to the accuracy or integrity of any part of the work are appropriately investigated and resolved.
